# Effect of 6-months of physical exercise on the nitrate/nitrite levels in hypertensive postmenopausal women

**DOI:** 10.1186/1472-6874-9-17

**Published:** 2009-06-19

**Authors:** Pedro R Zaros, Carla EM Romero Pires, Mauricio Bacci, Camila Moraes, Angelina Zanesco

**Affiliations:** 1 Department of Physical Education, Institute of Biosciences, University of Sao Paulo State (UNESP), Rio Claro (SP), Brazil; 2 Departmento of Biochemistry, University of Sao Paulo State (UNESP), Rio Claro (SP), Brazil; 3 Institute of Physical Activity and Sports, Cruzeiro do Sul University, Sao Paulo (SP) Brazil

## Abstract

**Background:**

Evidences have showed that the incidence of arterial hypertension is greater in postmenopausal women as compared to premenopausal. Physical inactivity has been implicated as a major contributor to weight gain and abdominal obesity in postmenopausal women and the incidence of cardiovascular disease increases dramatically after menopause. Additionally, more women than men die each year of coronary heart disease and are twice as likely as men to die within the first year after a heart attack. A healthy lifestyle has been strongly associated with the regular physical activity and evidences have shown that physically active subjects have more longevity with reduction of morbidity and mortality. Nitric oxide (NO) produced by endothelial cells has been implicated in this beneficial effect with improvement of vascular relaxing and reduction in blood pressure in both laboratory animals and human. Although the effect of exercise training in the human cardiovascular system has been largely studied, the majority of these studies were predominantly conducted in men or young volunteers. Therefore, the aim of this work was to investigate the effects of 6 months of dynamic exercise training (ET) on blood pressure and plasma nitrate/nitrite concentration (NOx^-^) in hypertensive postmenopausal women.

**Methods:**

Eleven volunteers were submitted to the ET consisting in 3 days a week, each session of 60 minutes during 6 months at moderate intensity (50% of heart rate reserve). Anthropometric parameters, blood pressure, NOx^- ^concentration were measured at initial time and after ET.

**Results:**

A significant reduction in both systolic and diastolic blood pressure values was seen after ET which was accompanied by markedly increase of NOx^- ^levels (basal: 10 ± 0.9; ET: 16 ± 2 μM). Total cholesterol was significantly reduced (basal: 220 ± 38 and ET: 178 ± 22 mg/dl), whereas triglycerides levels were not modified after ET (basal: 141 ± 89 and ET: 147 ± 8 mg/dl).

**Conclusion:**

Our study shows that changing in lifestyle promotes reduction of arterial pressure which was accompanied by increase in nitrite/nitrate concentration. Therefore, 6-months of exercise training are an important approach in management arterial hypertension and play a protective effect in postmenopausal women.

## Background

Epidemiological studies have shown that women life span is eight-year more compared to men. However, the incidence of cardiovascular disease in women increases dramatically after postmenopausal years, for instance, more women than men die each year of coronary heart disease (CHD) and are twice as likely as men to die within the first year after a heart attack. The risk factors of CHD are mainly related to dyslipidemia, arterial hypertension, physical inactivity, overweight, and diabetes mellitus [1]. Additionally, older women are at greater risk for weight gain and abdominal fat accumulation, a major component of metabolic syndrome. Physical inactivity has been implicated as a major contributor to overall weight gain and abdominal obesity in postmenopausal women [2,3].

A healthy lifestyle has been strongly associated with the regular physical activity. Evidences have shown that physically active subjects have more longevity with reduction of morbidity and mortality. Physical exercise prevents or reduces the deleterious effects of pathological conditions such as arterial hypertension, CHD, atherosclerosis, diabetes mellitus, and metabolic syndrome [4].

Nitric oxide (NO) produced by endothelial cells is the major regulator of the cardiovascular system acting on vascular basal tone, inhibiting platelet aggregation and smooth muscle proliferation [5]. The production of NO is regulated by an enzyme named NO synthase (NOS) which is activated by humoral (hormones, autacoids) and physical stimuli (shear stress) [6]. Vascular shear stress is a result of increased blood flow in the vessel wall and it is described as a potent stimulator of NO production from endothelium [7,8]. Physical exercise is a powerful stimulus to increase blood flow in vascular beds and consequently the beneficial effects of physical training on cardiovascular diseases are strongly associated with increase in NO production and/or its bioavailability either in human or laboratory animals [9-12]. Regarding the role of NO and physical training, the majority of the studies was predominantly conducted in men or young volunteers, but the effect of long-term of exercise training on the NO production and/or its bioavailability in postmenopausal women has not been investigated. Furthermore, evidences showed that the incidence of arterial hypertension is greater in postmenopausal women as compared to premenopausal age [11]. Therefore, the aim of this work was to investigate the effects of 6 months of dynamic exercise training on blood pressure and plasma nitrate/nitrite concentration in hypertensive postmenopausal women.

## Methods

### Participants screening

This study was approved by the Ethical Committee of the Institute of Bioscience from University of São Paulo State (UNESP/Rio Claro). The volunteers were recruited by advertisement in the Campus of UNESP and surrounding area. To be included in the study, postmenopausal women must be sedentary (regular aerobic exercise ≤ 2 sessions/wk and < 20 min/session, sedentary occupation); non-smoking; no diabetic (fasting glucose level < 110 mg/dl); an average systolic blood pressure from the two screening visits of 140–159 and a diastolic blood pressure of 90–99 mmHg (Stage 1 hypertension); and a medical condition that not exclude the exercise training. The exclusion criteria were subjects with cardiovascular disease (angina, valvular disease, stroke), diabetes, arthritis, alcohol consumption > 3 drinks per day, orthopedic conditions that affect the ability to participate in exercise program, neurological or psychiatric diseases, and pregnancy. To detect cardiovascular, pulmonary or other disease the volunteers were examined by a physician to selected the populations before the training test. A medical history, exercise training history and dietary history questionnaires were applied to the participants before the inclusion in this study. Eligible subjects were informed of the procedures and risks of the study and signed a written informed consent in accordance with the policies and ethical Committee of the Institute of Bioscience from UNESP. Eleven volunteers were included in this study.

### Anthropometric and cardiovascular parameters

Measurements of height were made using a clinical stadiometer in bare and body weight was measured with a digital calibrated precision scale (Thinner MS-7400, Fairlfiled NJ, USA). Body mass index (BMI) was determined by weight (Kg) divided by the square of the height in meters. Waist-hip ratio values (WHR) were also calculated. The anthropometric measurements were carried out at initial time and after 24 weeks of exercise training.

The volunteers were instructed not to exercise outside of the laboratory before blood pressure measurement. After 15 minutes of seated quiet rest, blood pressure was measured with aneroid sphygmomanometer (Tycos, Raleigh, NC) in 2 sessions. Resting heart rate was measured after 5 minutes in orthostatic position using a heart rate monitor (Polar A3, Kempele, Finland).

### Total cholesterol, triglycerides and glucose concentration

After 6 months of training exercise, venous blood samples were collected after 12 hours overnight fasting. Plasma was immediately separated by centrifugation (8,000 g) and Total cholesterol, triglycerides and glucose concentration were measured using specific kits (colorimetric method, Laborlab, São Paulo-SP, Brazil).

### Determination of plasma nitrite/nitrate (NO_x_^-^) levels

In order to evaluate the NO production, the plasma levels of nitrite and nitrate (NO_2_^-^/NO_3_^-^) were measured. Briefly, immediately after arterial blood collecting, the samples were centrifuged (8,000 g) for 10 min, and the resulting plasma supernatant was stored at -80°C. Plasma samples were ultrafiltered through microfilter cups (Microcon Centrifugal Filter Units, 10 kDa; Millipore, Bedford, MA, USA). The NO_x_^- ^concentration of the resulting filtrate solution was determined using a commercially available kit (Cayman Chemical, Ann Arbor, MI, USA) according to the manufacturer's instructions. This assay determines the total NO based on the enzymatic conversion of nitrate to nitrite by nitrate reductase. After the conversion, the spectrophotometric measurement of nitrite is accomplished by using the Griess Reaction. The resulting deep purple azo compound absorbs light at 540–550 nm.

### Exercise training

Each exercise session started and ended with appropriate warm-up and cool-down activities. To get familiarized to the cycle ergometer, in the first day of training, the participants performed an initial training session for 20 min increasing 10 min a day until 60 minutes of exercise training. The exercise sessions were supervised by personal trainer in all studied time. The participants were submitted to an aerobic exercise program on a cycle ergometer (Moviment, Jau SP, Brazil) in a quiet room with temperature at 25°C. Exercise sessions were performed for 3 days a week, 60 minutes each session, during 24 weeks with an intensity of 50% of heart rate reserve, according to the following equation [13].



### Statistics

Data were analyzed by using GraphPad Instat version 3.0 (GraphPad, San Diego CA, USA) using paired student *t *test. A *p *value < 0.05 was considered as statistically significant.

## Results

Neither BMI (baseline: 33 ± 1.4 and ET: 31 ± 1.5 Kg m^2-1^) nor WHR (baseline: 0.88 ± 0.06 and ET: 0.86 ± 0.05) were altered by exercise training program for 24 weeks. On the other hand, a significant reduction in both systolic (12%) and diastolic (10%) blood pressure values was seen after aerobic exercise training in hypertensive postmenopausal women. Heart rate was also reduced (approximately 8%) by physical training in this population. Data are summarized in Table 1.

**Table 1:** Anthropometric and cardiovascular parameters at initial time and after 6 months of aerobic exercise in hypertensive postmenopausal women

**Parameters**	**Initial time**	**After 24 weeks**
**Age (years)**	50 ± 4	50 ± 4
**Height (cm)**	161 ± 0.02	161 ± 0.02
**Weight (kg)**	86 ± 3.5	82 ± 3.3
**BMI (kg/m2)**	33 ± 6	32 ± 6
**WHR**	0.89 ± 0.06	0.87 ± 0.05
**SBP (mmHg)**	141 ± 10	123 ± 8*
**DBP **(**mmHg**)	90 ± 5	80 ± 5*
**HR (beats/min)**	83 ± 5	76 ± 6*

Data are mean ± S.E.M.

BMI: body mass index, WHR: waist-to-hip ratio, SBP: systolic blood pressures, DBP: diastolic blood pressure and HR: heart rate.

* *p *< 0.05, statistically different from initial time.

Total cholesterol concentration was significantly reduced after ET, approximately 20%. On the other hand, no changes were seen in the glucose and triglycerides levels. Plasma nitrite/nitrate concentration was markedly increased by 6-months of dynamic exercise training at moderate intensity in hypertensive postmenopausal women, approximately 60%. Data are summarized in Table 2.

**Table 2:** Lipid profile and glucose concentration at initial time and after 6 months of aerobic exercise in hypertensive postmenopausal women

**Parameters**	**Initial time**	**24 weeks**
Total cholesterol(mg/dl)	220 ± 38	178 ± 22*
Triglycerides (mg/dl)	141 ± 89	147 ± 88
Glucose (mg/dl)	99 ± 16	93 ± 16
Nitrite/Nitrate (mol/L)	10 ± 0.9	16 ± 2*

Data are means ± S.E.M for 11 volunteers.

*statistically different from initial time.

## Discussion

Our study shows that exercise training for 6 months promotes reduction in arterial blood pressure that is accompanied with increase in nitrite/nitrate concentrations in hypertensive postmenopausal women.

Physical exercise failed to provoke any alterations in BMI or waist-to-hip ratio in this particular population. The reason for that could be all the volunteers had no caloric intake restriction. Indeed, evidences show that effective loss of body weight is observed mainly when exercise training is associated with dietary restriction [14,15]. Another possibility to explain the lack of changing in BMI of the volunteers could be due to the increment of muscle mass in response to physical exercise training. Indeed, previous study showed that body fat percentage is decreased in trained women whereas the lean body mass is increased [16].

It is well established that exercise training promotes beneficial effect on cardiovascular system which has been associated with an improvement of the NO-cGMP pathway in the vasculature in both human and laboratory animals. Thus, exercise training is an important non-pharmacological tool in management arterial hypertension and atherosclerotic process [9,10]. Evidences have showed that the incidence of arterial hypertension is greater in postmenopausal women as compared to premenopausal. A number of factors can contribute to this increase in incidence of arterial hypertension in postmenopausal women including estrogen deficiency, alterations in lipid profile, weight gain, and decreases in physical activity during the menopause [2,3,17,18]. Indeed, a recent study has shown that estradiol deficiency tends to have a negative influence on NO-cGMP pathway in menopausal women, thus modification in lifestyle by physical exercise is an important approach to prevent cardiovascular complications in this population [19]. Our study shows that 6-month of dynamic exercise training was effective in lowering both systolic and diastolic blood pressure in a similar manner. This reduction was positively associated with increase in nitrite/nitrate concentration which reflects NO production. Considering that preventive actions to avoid high incidence of cardiovascular disease mainly arterial hypertension are extremely necessary for postmenopausal women, our results reinforce previous studies [20,21] and extend these findings showing that 6-months of exercise training (24 weeks) promotes a considerable increase in plasma nitrite/nitrate concentration (approximately 60%) as compared to studies where the physical exercise was applied for only 12 weeks with increment of 15–35% in plasma nitrite/nitrate concentration [20,21]. Thus, our findings show the improvement in cardiovascular system regulation is time-dependent of exercise training for this population.

Alterations in lipid profile play an important role for development of atherosclerosis and physical exercise has been successful in management lipid concentration in elderly volunteers [22]. In our studied population, the concentration of total cholesterol was slightly elevated according to the National Cholesterol Education Program's clinical practice guideline (> 200 mg/dl), whereas triglycerides level was at normal range (< 150 mg/dl). Aerobic physical training for 24 weeks provokes a significant decrease in total cholesterol concentrations in hypertensive postmenopausal women, but no changes in triglycerides levels were observed. As expected, physical exercise was effective to modify the elevated concentration of total cholesterol, but not triglycerides levels which is in agreement of the literature showing that alterations is observed when exist a previous abnormality.

## Conclusion

Our study shows that changing in lifestyle promotes reduction of arterial pressure which was accompanied by a marked increase in nitrite/nitrate concentration. Therefore, 6-months of exercise training are an important approach in management cardiovascular disease risk factors and play a protective effect in atherosclerosis in this particular population.

## Competing interests

The authors declare that they have no competing interests.

## Authors' contributions

PRZ carried out the training program studies, participated in the sequence alignment and drafted the manuscript. CEMRP helped carried out training program studies. MBJr helped with nitrite/nitrate analysis. CM in the design of the study and performed the statistical analysis. AZ conceived of the study, and participated in its design and coordination and helped to draft the manuscript. All authors read and approved the final manuscript.

## Pre-publication history

The pre-publication history for this paper can be accessed here:


